# Whole Genome-Sequencing and Phylogenetic Analysis of a Historical Collection of *Bacillus anthracis* Strains from Danish Cattle

**DOI:** 10.1371/journal.pone.0134699

**Published:** 2015-08-28

**Authors:** Sylviane Derzelle, Guillaume Girault, Branko Kokotovic, Øystein Angen

**Affiliations:** 1 University Paris-Est, Anses, Animal Health Laboratory, Maisons-Alfort, France; 2 National Veterinary Institute, Technical University of Denmark, Frederiksberg, Denmark; Loyola University Chicago, UNITED STATES

## Abstract

*Bacillus anthracis*, the causative agent of anthrax, is known as one of the most genetically monomorphic species. Canonical single-nucleotide polymorphism (SNP) typing and whole-genome sequencing were used to investigate the molecular diversity of eleven *B*. *anthracis* strains isolated from cattle in Denmark between 1935 and 1988. Danish strains were assigned into five canSNP groups or lineages, i.e. A.Br.001/002 (n = 4), A.Br.Ames (n = 2), A.Br.008/011 (n = 2), A.Br.005/006 (n = 2) and A.Br.Aust94 (n = 1). The match with the A.Br.Ames lineage is of particular interest as the occurrence of such lineage in Europe is demonstrated for the first time, filling an historical gap within the phylogeography of the lineage. Comparative genome analyses of these strains with 41 isolates from other parts of the world revealed that the two Danish A.Br.008/011 strains were related to the heroin-associated strains responsible for outbreaks of injection anthrax in drug users in Europe. Eight novel diagnostic SNPs that specifically discriminate the different sub-groups of Danish strains were identified and developed into PCR-based genotyping assays.

## Introduction

*Bacillus anthracis*, the etiological agent of anthrax, is a spore-forming, Gram-positive bacterium belonging to the *B*. *cereus* group. All mammals are known to be susceptible to anthrax, but the bacterium primarily affects herbivores, causing acute, often fatal disease. Anthrax is distributed throughout the world and still endemic in many countries, particularly in the developing world [1]. *B*. *anthracis* spends the majority of its life cycle as a dormant spore that can persist for long periods of time in many types of soil. Transmission to animals typically occurs by ingestion or inhalation of soil-borne spores [2–3].

Humans can be infected through contact with diseased animals or by exposure to spore-contaminated animal products (such as hides, wool, or meat). Depending upon the route of infection, anthrax could take one of several clinical forms with symptoms of different severity: gastrointestinal, cutaneous or inhalational [2]. Cutaneous anthrax is the most common and mild manifestation of the disease. However, a novel form of cutaneous anthrax, termed injectional anthrax, that is characterized by severe soft tissue infection has been recently described in heroin users [4]. The pathology of this can be particularly deadly. Pulmonary and gastrointestinal anthrax are less common but both forms can be particularly deadly if left untreated. Fatality rates can reach up to 90% for inhalational anthrax [5]. Prompt diagnosis has a major impact on the effectiveness of treatment.

*B*. *anthracis* is a monomorphic pathogen with extremely low genetic variability [6]. Due to this lack of diversity, only modern molecular characterization techniques with high discriminatory power are effective to differentiate strains. Microbiological forensics and epidemiological investigations increasingly rely on molecular markers, such as polymorphisms in DNA sequences, to obtain reliable information regarding the identification or source of a suspicious strain [7–10]. To identify a strain, comparative whole-genome sequencing and phylogenetic analysis are increasingly used to identify single-nucleotide polymorphisms (SNPs) specific for the particular strain [10–12]. The clonal nature of *Bacillus anthracis* makes SNPs ideal markers for subtyping of this pathogen and developing highly precise diagnostics assays. Identification of SNPs retrieved from compiled NGS sequences provides the highest possible resolution for molecular investigations [8].

By querying a large number of SNPs against collections of diverse strains, sets of canonical SNPs (canSNPs) that define major clades within the *B*. *anthracis* species have been identified and used for subdividing all isolates into three major lineages (A, B and C) and 13 lineages or groups [13]. In a continued effort to acquire and molecularly subtype isolates from around the world, other SNPs that define further the population structure of some lineages and groups or that lie on various branches of the *B*. *anthracis* SNP tree have also been identified [14–18].

In this report, canSNP analysis and whole-genome sequencing were used to subtype eleven cattle isolates from Denmark collected in the period 1935–1988. During the years 1900–1930, around 100 cases of bovine anthrax were reported annually in Denmark, declining to less than 10 cases annually after 1950. The last reported case of bovine anthrax in Denmark occurred in 1988. Comparative genomics were conducted using 41 additional strains belonging to various canSNP lineages to get insight into the worldwide phylogenetic placement of these Danish strains. Interestingly, those analyses resulted in the identification of two European strains as closely related to the heroin-associated *B*. *anthracis* outbreak strains as are the two Turkish isolates A0149 and A0264 [9], as well as two European strains belonging to the A.Br.Ames lineage, allowing the phylogenetical linkage of geographically disparate isolates and providing some clues to fill missing historical gap within the phylogeography of the A.Br.Ames lineage [14]. Novel SNPs that specifically discriminate the different sub-groups of Danish strains were identified and developed into PCR-based genotyping assays.

## Results and Discussion

### canSNP typing and whole genome sequencing

Eleven *B*. *anthracis* strains from the National Veterinary Institute's collection, Technical University of Denmark, were selected for this study (Table 1). All strains were first subjected to canonical single nucleotide polymorphism (canSNPs) analysis using high resolution melting [19] to determine their position within the established global *B*. *anthracis* phylogeny [9, 13, 20–21]. More than half of the *B*. *anthracis* isolates from Denmark clustered within the A.Br.001/002 group (n = 4) and its descended A.Br.Ames group (n = 2). The remaining isolates matched, respectively, the canSNP A.Br.008/011 sub-group (n = 2), A.Br.005/006 group (n = 2) and A.Br.Aust94 lineage (n = 1).

**Table 1:** Whole-sequenced strains and canSNP typing. The whole genome sequences of the Danish strains are available at: http://www.ebi.ac.uk/ena/data/view/PRJEB9705

**Strain**	**years**	**canSNP group**	**Sub-group**	Genome coverage*	**Sequencing depth**
K169/66	1966	A.Br.008/011	A08/D	99,991	137
K670/88	1988	A.Br Ames	A01/A	99,993	134
K35/88	1988	A.Br Ames	A01/A	99,993	136
K929	1966	A.Br.001/002	A02/B	99,991	132
1409	1974	A.Br Aust94	A.Br.013/015	99,951	126
K836	1968	A.Br 005/006	-	99,987	143
A	1935	A.Br.001/002	A01/C	99,997	144
B	1935	A.Br.001/002	A01/E	99,987	124
C	1960	A.Br.001/002	A02/A	99,899	130
D	1973	A.Br.008/011	A08/D	99,992	143
E	1937	A.Br 005/006	-	99,939	124
IEMVT89	unknown	A.Br.005/006	Africa	99,975	75

* Genome coverage are based on the Ames Ancestor genome size (5227419 bp).

All Danish strains were then characterized by paired-end whole genome sequencing, producing millions of reads per strain. After applying the quality filter of the Illumina base-calling pipeline, the filter-passed reads were aligned to the Ames Ancestor reference genome, resulting in more than a 50-fold sequencing depth on average and genome coverage higher than 99.6%.

### Extraction of whole strain-specific SNPs among *B*. *anthracis* strains

**Fig 1:**
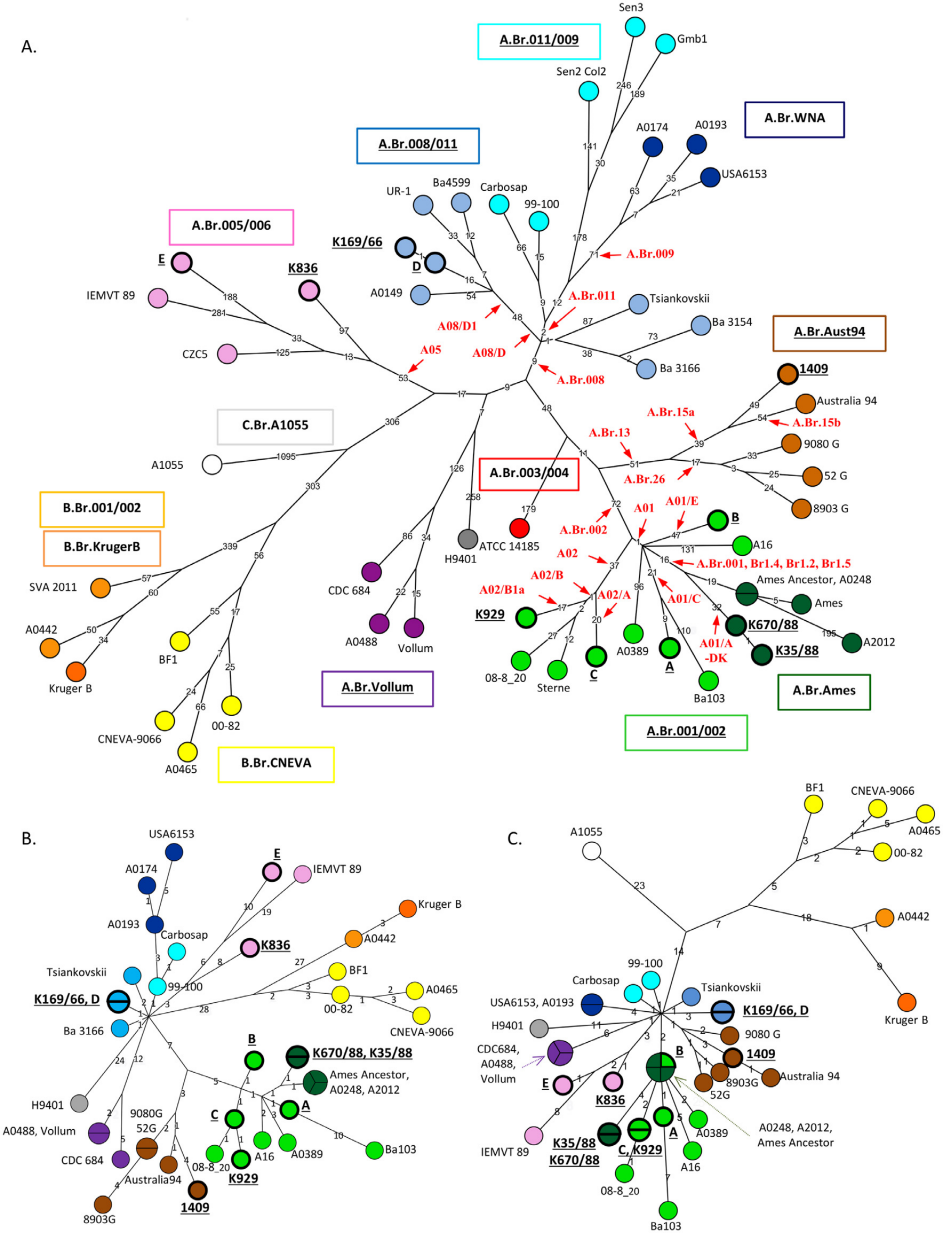
Position of the eleven Danish strains within the B. anthracis phylogenetic tree based on whole-genome SNP analysis. Minimum spanning tree based on 6596 chromosomal SNPs (A), 222 pXO1 SNPs (B) and 166 pXO2 SNPs (C). The 13 different canSNP groups are color-coded: C.Br.A1055 in white, B.Br.CNEVA in yellow, B.Br.001/002 and B.Br.Kruger in orange, A.Br.011/009 in light blue, A.Br.008/011 in blue, A.Br.WNA in dark blue, A.Br.005/006 in pink, A.Br.003/004 in red, A.Br.001/002 in green, A.Br.WNA in dark green, A.Br.Aust94 in brown and A.Br.Vollum in purple. The position of the 11 Danish isolates (in bold and underligned), the African IEMVT89 and 40 available whole genome-sequenced strains is marked. The length of each branch is proportional (logarithmic scale) to the number of SNPs identified between strains. Indicated in red are the position and name of some new or published SNPs specific to various canSNP groups: A05 (A.Br.005/006 group); A.Br.008 (A.Br.008/009 group); A08/D and A08/D1 (A.Br.008/011); A.Br.011 (A.Br.011/009); A.Br.009 (A.Br.WNA); A.Br.002 and A01 (A.Br.001/002 and A.Br.Ames); A02, A02/A, A02/B, A02/B1 (A.Br.001/002 subgroup A02); A.Br.001 and A01/A-DK (A.Br.Ames); A.Br.013, A.Br.015a, A.Br.15b, A.Br.026 (A.Br.Aust94). Based on a parsimony approach, the trees sizes are, respectively, 6730 (A), 227 (B) and 168 SNPs (C), i.e. containing approximately 1.9 (A), 2.2 (B) or 1.2 (C) % of homoplasia. Available from the Dryad Digital Repository, see S1 File.

Comparative analysis of the genomic sequences of these strains was carried out using genomic data of 41 additional *B*. *anthracis* strains of worldwide origin, 33 belonging to various lineages of clade A, seven of clade B and one of clade C (Table 2). A total of 6596 non-homoplasic chromosomal SNPs were identified and used to draw the phylogeny of the eleven Danish strains. Additional SNPs located on both pXO1 and pXO2 plasmids were also identified (i.e. 222 and 166 SNPs for pXO1 and pXO2, respectively). Fig 1 illustrates the minimum spanning tree (MST) generated by the chromosomal and plasmidic whole-genome SNPs analysis.

**Table 2:** Whole-genome sequences from public database used in this study.

**Strain**	**Country**	**canSNP**	**Accession number**
Ames Ancestor	USA	A.Br.Ames	NC_007530.2
A2012	USA	A.Br.Ames	AAAC00000000.1
Ames	USA	A.Br Ames	NC_003997.3
A0248	USA	A.Br Ames	NC_012659.1
Sterne	South Africa	A.Br 001/002	AE017225.1
A0389	Indonesia	A.Br 001/002	ABLB00000000.1
BA103	Japan	A.Br 001/002	DRR000183 (SRA)
A16	China	A.Br 001/002	CP001970.1
ATCC14185	Israel	A.Br 003/004	AZQO00000000.1
CZC5	Zambia	A.Br 005/006	BAVT00000000.1
Tsiankovskii-1	Soviet Union	A.Br 008/011	ABDN00000000.2
Ba3154	Bulgaria	A.Br 008/011	ANFF00000000.1
Ba3166	Bulgaria	A.Br 008/011	ANFG00000000.1
Ba4599	Scotland	A.Br 008/011	AGQP00000000.1
UR-1	Germany	A.Br 008/011	ALNY00000000.1
A0149	Turkey	A.Br 008/011	SRX562299
Carbosap	Italy	A.Br 011/009	ANAO00000000.1
Sen2Col2	Africa	A.Br 011/009	CAVC000000000.1
Sen3	Africa	A.Br 011/009	CAVD000000000.1
Gmb1	Africa	A.Br 011/009	CAVE000000000.1
Australia 94	Australia	A.Br.Australia94	AAES00000000.1
9080 G	Georgia	A.Br.Australia94	AZUE00000000.1
52 G	Georgia	A.Br.Australia94	AZUF00000000.1
8903 G	Georgia	A.Br.Australia94	AZUD00000000.1
CDC684	USA	A.Br.Vollum	NC_012581.1
H9401	Korea	A.Br.H9401	NC_017729.1
A0488	UK	A.Br.Vollum	ABJC00000000.1
Vollum	UK	A.Br.Vollum	AAEP00000000.1
USA6153	USA	A.Br.WNA	AAER00000000.1
A0174	Canada	A.Br WNA	ABLT00000000.1
A0193	USA	A.Br WNA	ABKF00000000.1
99–100	France	A.Br.011/009	JHDR00000000
08-8/20	France	A.Br.001/002	JHCB00000000
00–82	France	B.Br.CNEVA	JHDS00000000
CNEVA-9066	France	B.Br.CNEVA	NZ_AAEN00000000.1
A0465	France	B.Br.CNEVA	NZ_ABLH00000000.1
BF1	Germany	B.Br.CNEVA	AMDT00000000.1
Kruger B	South Africa	B.Br.Kruger	AAEQ00000000.1
A0442	South Africa	B.Br.001/002	ABKG00000000.1
SVA 11	Sweden	B.Br.001/002	CP006742.1
A1055	USA	C.Br.A1055	AAEO00000000.1
AH820	Norway	*Bacillus cereus*	NC_011773.1

Additional rooted phylogenetic trees were also generated using the *B*. *cereus* AH820 strain as outgroup and a final dataset of 29906 SNPs (S1 Fig). Phylogenetic relationships of the Danish strains were confirmed using Maximum Likelihood, Maximum Parsimony, Neighbor-Joining and UPGMA methods (S1 Fig).

### A.Br.001/002 phylogenetic analysis

The resulting whole-genome SNP phylogenetic tree revealed two genetic groups, A01 and A02, along the A.Br.001/002 group (Fig 1) and at least five novel branches (Branches a to e) within the A01 sub-group terminating in the Ames ancestor genome. The A01 sub-group (also termed “Ames sub-group”) radiates very shortly after the A01-A02 divergence (1 SNP) as previously described [20]. Four Danish strains clustered into A01and were resolved into three out of the five identified A01 branches. Branch a matches the A.Br.Ames lineage (strains Ames Ancestor, Ames, A2012 and A0248), including the clonal Danish isolates K670/88 and K35/88, differing by a single SNP (Fig 1). We identified 32 SNPs specific to these strains isolated in South Jutland in 1988. The other Danish A01-affiliated strains were grouped into Branch c (strain A) or Branch e (strain B). The B isolate was unrelated to any other A.Br.001/002 strain analyzed in this study. Strain A belonged to the same genetic group as the Japanese Ba103 strain [16], as observed on both plasmidic and chromosomal SNP trees. Strains isolated in China (A16) [22] or Indonesia (A0389) [13] constituted, respectively, branches d and b. Two additional Danish isolates (K929 and C) clustered within the A02 sub-group (also termed “Sterne sub-group”) that includes the terminal reference Sterne strain and French A.Br.001/002 strains like 08–8_20 that were recently collected in the Doubs *department* [17].

To better position the Danish Ames-like strains within the A.Br.Ames lineage, the two strains were further genotyped using previously published Ames-specific SNP assays [14]. Strains K670/88 and K35/88 share the derived allele with Ames genetic relatives at four out of 29 described SNPs (i.e. Br1.4, Br1.2, Br1.21, Br1.5). Ames-like isolates from China and Texas share common ancestors that originated in inner Mongolia, China [14]. It is believed that an Ames-like isolate was probably introduced in the USA by early settlers or traders from Europe, during the early to mid-1800s. *B*. *anthracis* was introduced into the Gulf Coast through New Orleans and or Galveston. The disease became established along the coastal regions and then became endemic to the regions of Texas where cattle are currently farmed [14]. However, no isolate from this lineage was ever found in Europe to support this hypothesis, leaving a historical gap within the phylogeography of the Ames lineage that this report has filled.

### A.Br.008/009 phylogenetic analysis

The two Danish A.Br.008/011 strains, collected in 1966 (K169/66) and 1973 (D), were found to be highly clonal, with only one chromosomal SNP to discriminate both strains (Fig 1). They were phylogenetically related to heroin-associated strains (Ba4599 and UR-1) isolated in 2009/2012 in Europe [10, 23]. Outbreaks of injection anthrax in heroin users have been going on in Western Europe since at least the year 2000, with a highly probable similar source of contamination which might be still active [10, 24–25]. The first case was reported from Norway in 2000 [26] and in 2009–2010, a total of 119 similar cases occurred in Scotland of which 47 were laboratory-confirmed anthrax cases [24]. Further five cases were reported from England and three from Germany. The most recent outbreak started in 2012. At least 13 cases occurred in Germany, France, Denmark and United Kingdom, resulting in five fatalities [24].

SNP assays (SNP1173928 and SNP1053700) used for bioforensic genotyping of heroin-associated *Bacillus anthracis* strains were performed [10, 25]. The Danish strains do not share the allelic states of heroin-associated strains but the presence of five further SNPs (SNP5013862, SNP1967560, SNP1530761, SNP3287006 and SNP3836105) with increasing specificity for the ‘heroin isolates’ were confirmed. It can be concluded that strains K169/66 and D are as closely related to Ba4599 and UR-1 as is the closest relative identified so far, the Turkish A0149 strain [9]. This study demonstrated that near relatives to heroin-associated strains were already found in Europe before the first report of injection anthrax outbreak in 2000 in Norway. The heroin-associated strains circulating in Europe are supposed to originate from the Middle East [9]. The real origin where the heroin’s contamination comes from is still a matter of speculation. The A.Br.008/011 sub-group has been widely successful in distribution across Europe and parts of Asia.

### A.Br.005/006 phylogenetic analysis

Two phylogenetically unrelated Danish strains, i.e. E and K836, belong to the A.Br.005/006 canSNP group that is ecologically established in Africa (Fig 1). Unlike A.Br.001/002 and A.Br.008/009, this group appears not to be widely distributed in Europe and might therefore be associated with trade of animal products from Africa contaminated with anthrax spores. But only a few A.Br.005/006 strains are available for comparison and very little information is known regarding the molecular genotypes of isolates present in Africa, especially from central and western regions. Molecular subtyping of additional *B*. *anthracis* isolates from this group is therefore necessary to draw any conclusion on the dissemination of such strains from Africa to Europe. Detailed phylogeography of this lineage has not been previously described. The present whole-genome SNP analysis revealed that the Danish E strain and the African IEMVT89 strain share common ancestors, although a long evolutionary time period separate both isolates (the distance to a possible common ancestor is 188 and 281 SNPs, respectively).

### A.Br.Aust94 phylogenetic analysis

The last Danish genome (strain 1409) was placed within the A.Br.Aust94 genetic group. To obtain a more accurate placement of this strain along the *B*. *anthracis* SNP tree, we further screened it with a few recently published A.Br.Aust94-specific SNP assays [19, 21]. Strain 1409 possesses a derived allele for SNP A.Br.015a (SNP 182717) but does not share the SNP A.Br.015b (SNP 317219) allele with the reference Australia94 genome. It can be therefore concluded that strain 1409 splits out from the branch leading to the terminal Australia94 strain, at a short distance from the basal A.Br.015/016 sub-group (characterized by a derived nucleotide for both A.Br.015a and A.Br.015b SNPs). This genetic sub-group may not be ecologically established in Europe. Rather, the presence of such strain in Denmark is likely due to importations of contaminated animal products from Asia or Africa. Additional phylogeography studies of the A.Br.Aust94 group are needed to be able to identify this source.

### Specific SNP discrimination genotyping assay

Eight novel SNPs that discriminate the different sub-groups containing Danish strains were developed into HRM assays (Table 3). Six assays targeted the A.Br.001/002 group (i.e. A01/A, A01/C, A01/E, A02/A, A02/B and A02/B1a), two others the A.Br.008/011 sub-group including the heroin-associated strains and their near relatives (i.e. A08/D and A08/D1) (Table 3, Fig 1). These novel diagnostic assays were validated across 250 *B*. *anthracis* DNAs in collection. The two expected alternate alleles exhibited distinct melting curves and melting temperatures, allowing unambiguous discrimination of the Danish strains within their respective genetic sub-groups (data not shown).

**Table 3:** Specific canSNPs and primer sequences used for HRM analysis.

**canSNP**	position*	**SNP**	**mean Tm (°C)**	**PCR (bp)**	**Forward primer (5’-3’)**	**Reverse primer (5’-3’)**
A01/A-DK	4049602	T/C	75.1/75.9	59	TCCTATGCGTCCTTCTTTACTCA	CAATGAAGAACCAAGACCAAGC
A01/E	4407325	G/A	78.3/79.0 Dk8A01	83	GAAGTTTCTGACGGCTTAATGG	CGTTTTTGTACTAATGGTACTTCTTCTG
A01/C	2045104	G/A	75.0/75.8	69	GCGGATAGTCGTGAAATGTTAAA	GAGATACCCTCGTCCTGGTACAT
A02/A	4961938	G/A	79.5/80.3	75	TTGTTAACGCAACATCTCTACGC	CATTTAATACGCCACCAATTACG
A02/B	513052	C/T	72.5/73.4	61	AAAATAAAAACCAGAGAGCGAATATC	CTCCTGTAACGCCTACCAAAGAT
A02/B1a	3668372	A/G	76.5/77.2	76	TCGTAAACGAACTCATTAACTGC	GCGCTAAAATCTTTAGAAGGAACA
A08/D	108749	C/A	77.1/78.1	67	CAAGATGTGTTCATGGGAGATTTC	CTGCACCGTTAATTACGAATGTT
A08/D1	1530761	A/T	75.7/76.4	56	TTGAAACAATAGGGGCATTAGG	TTCCGCTACTGCTTCTTACACA

* localisation on the Ames Ancestor chromosome (GenBank accession no. AE017334.2).

### Conclusions

In this study, the A.Br.001/002 canSNP group has been shown to be predominant in Denmark. This canSNP group accounts for a significant part of the European *B*. *anthracis* population in Belgium, the United Kingdom, the Netherlands and northeastern France [8, 17]. Other canSNP groups, including the successful transeurasian A.Br.008/011 sub-group, the African A.Br.005/006 group and the Asian A.Br.Aust94 lineage, are also present in the country and contribute to the overall genetic diversity found in Europe.

This report illustrates the need to acquire and genotype isolates from around the world to generate hypotheses about epidemiological linkage of geographically disparate isolates and global patterns of dispersal of the anthrax agent. Intensive national sampling studies of *B*. *anthracis* collections are invaluable for identifying the real origin of a strain so that accurate assignments can be made.

## Materials and Methods

### DNA extraction

All strains were grown on Colombia agar plates (Difco) added 5% bovine blood at 37° overnight. Surface growth was harvested and DNA extraction was performed using Genomic Tips 100/G (Qiagen) according to the manufacturer’s recommendations.

Viability testing was systematically performed before DNA was taken out of the BSL-3 facility. All *B*. *anthracis* manipulations were performed in a biosafety level 3 laboratory using a class II type A2 biosafety cabinet.

### CanSNP typing by HRM

CanSNP analysis was performed as previously described [19]. This technique categorizes isolates into one of 12 sub-lineages (C.Br.A1055, B.Br.CNEVA, B.Br.KrugerB, A.Br.Vollum, A.Br.Aust94, A.Br.Ames, A.Br.WNA, B.Br.001/002, A.Br.005/006, A.Br.003/004, A.Br.001/002, A.Br.008/009). Additional canSNP that provides resolution within the A.Br.008/009, A.Br.001/002, A.Br.Ames and A.Br.Aust94 groups were also used [10, 14, 18, 20–21].

High Resolution Melting (HRM) assays were designed using Primer 3^+^ software [27]. The primers sequences used are listed in Table 3. Amplification was performed on the ViiA7Real-Time PCR System (Life Technologies) using the LightCycler 480 High Resolution Melting Master Mix (Roche Diagnostics). The reaction mixture consisted of 0.2 μM of each primer, 1×LightCycler 480 HRM master mix and 2.5 mM MgCl2 in a 10-μl final volume. The following parameters were used: 10 min at 95°C were followed by 35 cycles consisting of 10 s at 95°C, 10 s at 58°C and 10 s at 72°C. Samples were next heated to 95°C for 30 s, cooled down to 65°C for 1 min and heated from 65°C to 88°C at a rate of 1°C/s with 25 acquisitions/°C. HRM data were analyzed by the ViiA7Software (version 1.2.1).

### Draft whole genome sequencing (WGS) and data analysis

Isolates were subjected to paired-end whole genome sequencing on the Illumina HiSeq2000 platform (paired-end data of 2x100pb) (Illumina Inc., San Diego, CA, USA).

Ames Ancestor [GenBank:AE017334.2] was used as the reference genome for assembly. Ames Ancestor plasmid pXO1 [GenBank:AE017336.2] and pXO2 [GenBank:AE017335.3] were used as references for plasmids assembly. Short reads data sets were exported on the FastQ format and mapped to the Ames Ancestor genome and both pXO1 and pXO2 plasmidic sequences using BioNumerics version 6.6 (Applied Maths, Belgium) and Power assembler module asking for a similarity of at least 90%. A set of SNPs was deduced for each genome sequence data using BioNumerics Chromosome Comparisons module. Individual lists were compiled and data filtered to remove SNP positions at which one or more isolate displayed an ambiguous residue call or missing data. Ribosomal operons and VNTR loci were also excluded from the analysis, as well as contiguous SNPs (using a window-frame of 10 bp).

### Whole genome phylogenetic analysis

The genomic sequences for 40 available *B*. *anthracis* strains used for comparison can be found in the NCBI microbial genome website at http://www.ncbi.nom.nih.gov.

Unrooted minimum spanning trees were drawn in BioNumerics version 6.6 (Applied Maths, Belgium) by using the filtered whole genome sequencing SNP data as input and default settings (Fig 1). Nodes were numbered by BioNumerics Additional rooted trees, including Bootstrap values, were next generated in MEGA6.0 [28] using the *B*. *cereus* AH820 strain as outgroup and a final dataset of 29906 SNPs (S1 Fig). Phylogenetic relationships were inferred using four distinct methods, i.e. Maximum Likelihood, Maximum Parsimony, Neighbor-Joining and UPGMA. All positions containing gaps and missing data were eliminated. The Maximum Likelihood analysis was based on the Jukes-Cantor model. Initial tree(s) for the heuristic search were obtained by applying the Neighbor-Joining method to a matrix of pairwise distances estimated using the Maximum Composite Likelihood approach. The Maximum Parsimony tree was obtained using the Subtree-Pruning-Regrafting algorithm with search level 1 in which the initial trees were obtained by the random addition of sequences (10 replicates). For both the neighbor-joining and UPGMA methods, the evolutionary distances were computed using the Maximum Composite Likelihood method.

## Supporting Information

S1 Fig Evolutionary analyses of 52 strains of *B*. *anthracis* based on 29906 chromosomal SNPs. Phylogenetic relationships were inferred using the Maximum Likelihood method (A), the Maximum Parsimony method (B), the Neighbor-Joining method (C) and the UPGMA method (D). Bootstrap values (100 iterations) higher than 70% are shown next to the branches. All evolutionary analyses were conducted in MEGA6 [28]. The maximum likelihood tree with the highest log likelihood (0.000) is shown in A. Tree #1 out of 2 most parsimonious trees (length = 7469, consistency index = 0.815, retention index = 0.947) is shown in B. The optimal neighbour-joining tree with the sum of branch length = 0.251 is shown in C. The optimal UPGMA tree with the sum of branch length = 0.239 is shown in D. The later tree is drawn to scale, with branch lengths in the same units as those of the evolutionary distances used to infer the phylogenetic tree. Available from the Dryad Digital Repository, see S1 File.
(TIF)

S1 File Dryad Digital Repository URL. (DOCX)

S1 Table Published lineage- or group-specific canSNPs used in this study. (DOCX)
