# Supplementary figures and images for: Whole Genome-Sequencing and Phylogenetic Analysis of a Historical Collection of Bacillus anthracis Strains from Danish Cattle
Source: PLoS One. 2015 Aug 28;10(8):e0134699. doi: 10.1371/journal.pone.0134699 (PMC4552859; doi:10.1371/journal.pone.0134699)

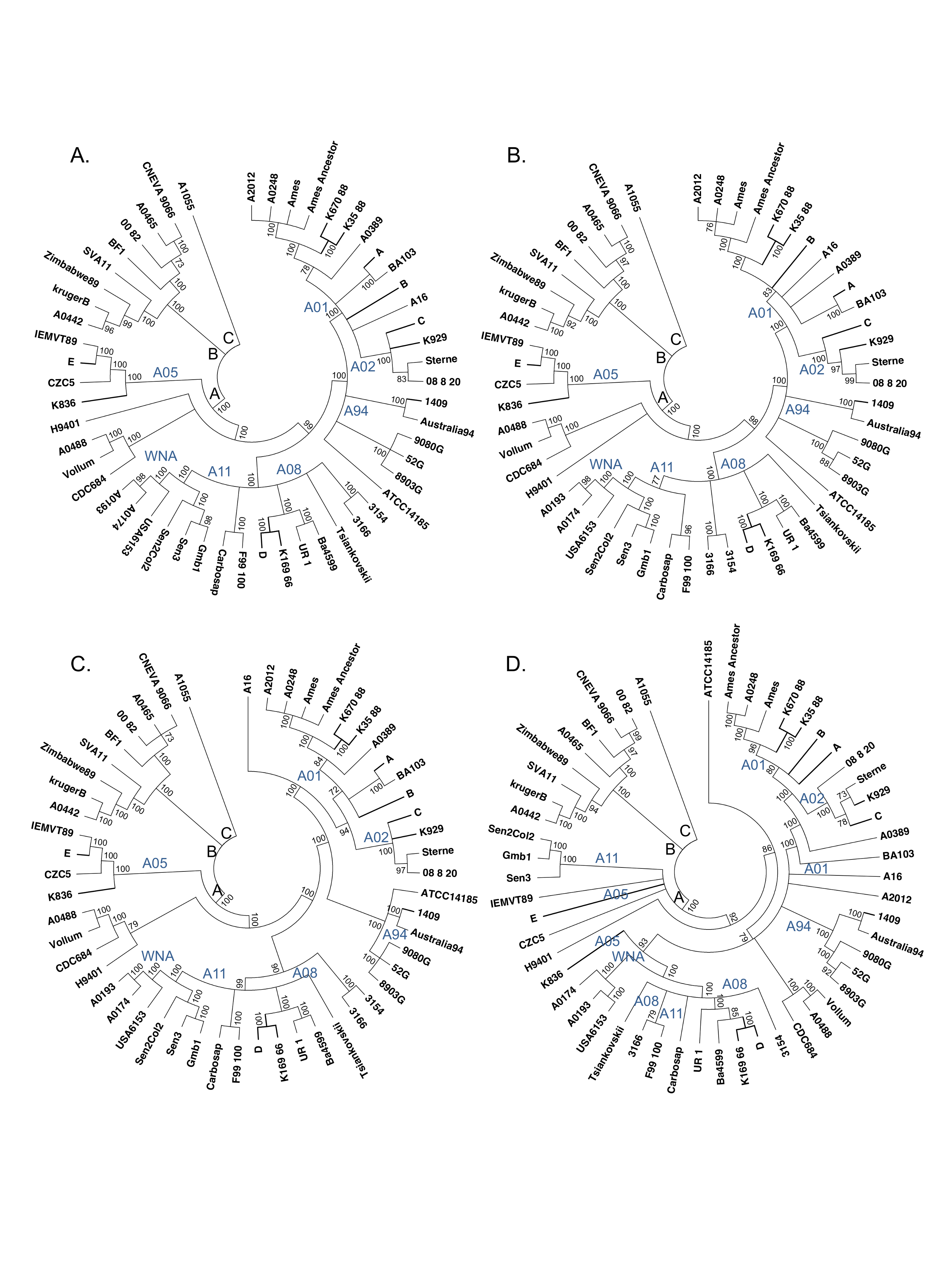

Supplement: S1 Fig — Phylogenetic relationships were inferred using the Maximum Likelihood method (A), the Maximum Parsimony method (B), the Neighbor-Joining method (C) and the UPGMA method (D). Bootstrap values (100 iterations) higher than 70% are shown next to the branches. All evolutionary analyses were conducted in MEGA6 [28]. The maximum likelihood tree with the highest log likelihood (0.000) is shown in A. Tree #1 out of 2 most parsimonious trees (length = 7469, consistency index = 0.815, retention index = 0.947) is shown in B. The optimal neighbour-joining tree with the sum of branch length = 0.251 is shown in C. The optimal UPGMA tree with the sum of branch length = 0.239 is shown in D. The later tree is drawn to scale, with branch lengths in the same units as those of the evolutionary distances used to infer the phylogenetic tree. Available from the Dryad Digital Repository, see S1 File. (TIF) [file pone.0134699.s001.tif]
