# Supplementary material for: Whole Genome-Sequencing and Phylogenetic Analysis of a Historical Collection of Bacillus anthracis Strains from Danish Cattle
Source: PLoS One. 2015 Aug 28;10(8):e0134699. doi: 10.1371/journal.pone.0134699 (PMC4552859; doi:10.1371/journal.pone.0134699)
Supplement: S1 File — (DOCX) [file pone.0134699.s002.docx]

Fig. 1 and S1 Fig. are available from the Dryad Digital Repository: <http://dx.doi.org/10.5061/dryad.0h82m>
