# Supplementary material for: Whole Genome-Sequencing and Phylogenetic Analysis of a Historical Collection of Bacillus anthracis Strains from Danish Cattle
Source: PLoS One. 2015 Aug 28;10(8):e0134699. doi: 10.1371/journal.pone.0134699 (PMC4552859; doi:10.1371/journal.pone.0134699)
Supplement: S1 Table — (DOCX) [file pone.0134699.s003.docx]

**Supplementary data**

**Table S1. Published lineage- or group-specific canSNPs used in this study**

| canSNP | position* | SNP | Target lineage | Reference |
| --- | --- | --- | --- | --- |
| A.Br.13 | 3101332 | A to G | A.Br.Aust94 | 20 |
| A.Br. 15a | 182717 | G to A | A.Br.Aust94 | 20 |
| A.Br. 15b | 317219 | A to G | A.Br.Aust94 | 20 |
| A01 | 515111 | A to G | A.Br.001/002, A.Br.Ames | 19 |
| A02 | 240050 | T to C | A.Br.001/002 | 19 |
| Br.1.4 | 200731 | T to C | A.Br.Ames | 13 |
| Br.1.2 | 182106 | T to C | A.Br.Ames | 13 |
| Br.1.5 | 243867 | T to C | A.Br.Ames | 13 |
| ABr001 | 182106 | T to C | A.Br.Ames | 12 |
| A05 | 405303 | C to T | A.Br.005/006 | 19 |
| unnamed | 5013862 | T to A | A.Br.008/011 heroin-like | 9,24 |
| unnamed | 1967560 | C to A | A.Br.008/011 heroin-like | 9,24 |
| unnamed | 1530761 | A to T | A.Br.008/011 heroin-like | 9,24 |
| unnamed | 3287006 | C to A | A.Br.008/011 heroin-like | 9,24 |
| unnamed | 3836105 | T to C | A.Br.008/011 heroin-like | 9,24 |
| unnamed | 1053700 | A to G | A.Br.008/011 heroin | 9,24 |
| unnamed | 1173928 | G to C | A.Br.008/011 heroin | 9,24 |

* localisation on the Ames Ancestor chromosome (GenBank accession no. AE017334.2).
